# The use of normal forms for analysing nonlinear mechanical vibrations

**DOI:** 10.1098/rsta.2014.0404

**Published:** 2015-09-28

**Authors:** Simon A. Neild, Alan R. Champneys, David J. Wagg, Thomas L. Hill, Andrea Cammarano

**Affiliations:** 1 Faculty of Engineering, University of Bristol, Bristol BS8 1TR, UK; 2 Department of Mechanical Engineering, University of Sheffield, Sheffield S1 3JD, UK

**Keywords:** nonlinear dynamics, normal forms, modal analysis, cable vibration

## Abstract

A historical introduction is given of the theory of *normal forms* for simplifying nonlinear dynamical systems close to resonances or bifurcation points. The specific focus is on mechanical vibration problems, described by finite degree-of-freedom second-order-in-time differential equations. A recent variant of the normal form method, that respects the specific structure of such models, is recalled. It is shown how this method can be placed within the context of the general theory of normal forms provided the damping and forcing terms are treated as unfolding parameters. The approach is contrasted to the alternative theory of nonlinear normal modes (NNMs) which is argued to be problematic in the presence of damping. The efficacy of the normal form method is illustrated on a model of the vibration of a taut cable, which is geometrically nonlinear. It is shown how the method is able to accurately predict NNM shapes and their bifurcations.

## 1. Introduction: nonlinear normal modes and normal forms

The problem addressed here is how to extend the well-established notion of *normal modes* of linear vibration systems to nonlinear systems in a mathematically consistent way that also allows for practical implementation. In recent years, there has been a lot of research related to the concept of a so-called ‘*nonlinear normal mode*’ (NNM). We shall not go into the complete theory here, but refer instead to Kerschen *et al.* [1] and Peeters *et al.* [2]) and Avrimov & Mikhlin [3] for surveys of the state of the art. As we shall explain, this concept is of limited use when forcing and damping are present, a restriction that is not shared by the more general concept of the *normal form* of a nonlinear system near an equilibrium [4–6]. At present though, the application of normal form analysis to structural vibration systems [7,8], described in more detail in §3 below, is rather technical and not widely adopted. It is also not clear how these structural vibration normal forms relate to the generic theory of normal forms as treated in Murdock [6]. Thus, the aim of this paper is to explain the normal form theory for structural vibrations in a more rigorous and historical context, and to show its relevance for a variety of practical problems.

**Figure 1.**
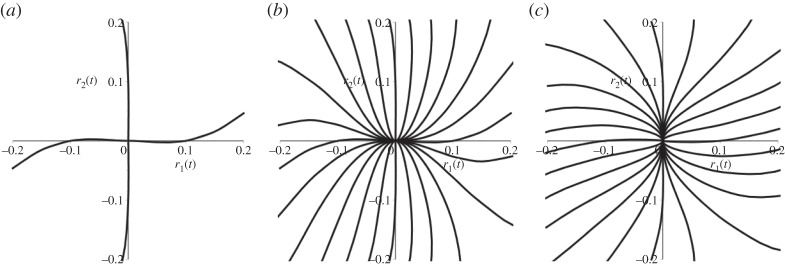
Illustrating the limitation of the NNM concept in the presence of damping. Computations are for the unforced two-degree-of-freedom system whose radial parts are written in polar coordinates as 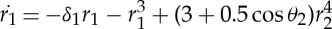, 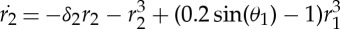, where (*a*) *δ*_1_=*δ*_2_=0, (*b*) *δ*_1_=0.1, *δ*_2_=0.05 and (*c*) *δ*_1_=0.05,*δ*_2_=0.1. We show projections, ignoring the angular motion, of the invariant manifolds of the system that are tangent to the linear eigenspaces *r*_1_=0 and *r*_2_=0.

Consider mechanical structures: the dynamics can be modelled as
1.1
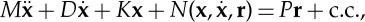
where 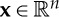 represents the displacements of the *n* degrees of freedom of the structure, *M*, *D* and *K* are, respectively, mass, damping and stiffness matrices and *N* represents nonlinearity, which is assumed to be sufficiently smooth. The right-hand side represents the forcing on the system 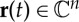 and can be written as a sum of e^i*Ω*_*i*_*t*^ terms. The concept of the NNM applies well to the case where there is no forcing or damping, so that *P*=*D*=0 in (1.1) and *N* is a pure function of **x**. In this case, Shaw & Pierre [9,10] argued that an NNM is just an invariant manifold composed of periodic solutions, whose frequency in the limit of amplitude tending to zero is the same as the linear mode. Here, they can appeal to the Liapunov Centre Theorem that guarantees that such a manifold must exist. Attempts have been made to extend this definition to include damping. Here, things are more troublesome because the Liapunov centre theorem no longer applies and, in general, one should not expect there to be small periodic solutions. Nevertheless, one can still appeal to the theory of invariant manifolds, but unfortunately such manifolds will be non-unique in general and will depend crucially on the relative size of the damping in each mode; see figure 1 illustrating this point for a general two degrees of freedom system. Figure 1*a* illustrates NNMs the case of zero damping. These manifolds which are shown as projections onto a radial coordinate are composed entirely of periodic orbits, as given by the Liapunov centre theorem. By contrast, figure 1*b*,*c* shows damped cases where the damping coefficient is greater in either the first or the second mode. In each case, only the most strongly damped mode has a unique invariant manifold tangent to it, with solutions in the manifold now composed of decaying oscillations. For the more weakly damped mode, there are now infinitely many such invariant manifolds and there is no uniquely distinguished one. In general, a tiny perturbation in initial condition will lead to a very different manifold if one computes backwards in time.

In the presence of general forcing, there is no guarantee that these invariant manifolds persist. Instead, what one needs is a *calculation tool*; a way of simplifying the system that respects normal modal analysis in the limit of small amplitude response, but for moderate amplitude also allows for the inclusion of only the necessary nonlinear effects. This is precisely what the theory of normal forms does. A concise description of that theory forms the subject of §2.

The link between normal form theory and NNMs was pointed out by Montaldi *et al.* [11], who showed that NNMs can be constructed directly, as a consequence of the Birkhoff normal form (§2) for *undamped* systems written in Hamiltonian coordinates. More general normal analysis was carried out on various structural systems in the late 1970s and early 1980s by Holmes and co-workers, see Guckenheimer & Holmes [4] and references therein. Jezequel & Lamarque [7] showed how forcing and damping can be incorporated in the approximation of normal forms for systems equivalent to (1.1), but written in first-order, i.e. state-space, form. Their method is essentially a hybrid between normal form reduction and the harmonic balance method. Direct harmonic balance and other asymptotic methods, such as multiple scales analysis have been applied by many authors to derive approximations to systems of the form (1.1) [12].

Recently, Neild & Wagg [8] have extended the work of Jezequel & Lamarque [7] to deal directly with second-order systems of the form (1.1); see §1 for the details. One purpose of this paper is to survey their method and to demonstrate its power through applications. Primarily though, we shall show how the second-order method can be recast using the general theory of normal forms, where forcing and damping terms can be treated as specific forms of unfoldings. One does not need to make the additional approximations inherent in the harmonic balance method when deriving the normal form itself. Application of the harmonic balance method to the derived normal form, though, is shown to lead to remarkably powerful predictions for resonant responses, leading to so-called *backbone curves* in structural analysis, for mode switching in complex multimodal responses and for analytically finding NNMs.

The rest of this paper is outlined as follows. In §2, we describe the historical context of normal form transformations. Section 3 goes on to apply these concepts to engineering vibration problems of the form (1.1). In particular, we show how the Neild & Wagg method can be thought of as the choice of a particular type of normal form. We also explain how to use this method to predict resonant responses, by interpreting the normal form in the context of harmonic balance. Section 4 demonstrates the theory through application to a particular model of a taut cable. The power of the method is illustrated by showing how it can predict secondary bifurcations corresponding to switches in mode shapes. Finally, §5 draws conclusions and points to avenues of future work.

## 2. Historical background to normal form analysis

Much of our modern-day understanding of dynamical systems can be traced to the work of the French genius Henri Poincaré and the geometric theory he introduced [13] to understand systems like the three-body problem in celestial mechanics; see, for example, Barrow-Green [14], Diacu & Holmes [15], Verhulst [16] for historical reviews. His key insight was to work on approximating the system rather than producing series solutions for individual trajectories.

### (a) Birkhoff normal form

The original context for normal forms was that of conservative systems written in Hamiltonian form. Consider a Hamiltonian system in 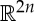 close to an elliptic equilibrium point, written in canonical form
2.1
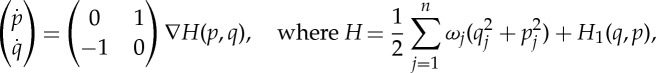
in which 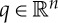 represents the column vector of positions (*q*_1_,*q*_2_,…,*q*_*n*_)^T^, *p* represents the momenta (*p*_1_,*p*_2_,…,*p*_*n*_)^T^ and *H*_1_ contains all higher-power terms, starting with cubic. For the purposes of this paper, we shall assume that all vector fields are analytic (infinitely smooth) and we shall suppress any parameter dependence for the time being. Without the nonlinear terms, the system (2.1) is easily solvable and decomposes into quasi-periodic motion with independent frequencies *ω*_*j*_, *j*=1,2,…,*n*. That is, in each degree-of-freedom, the motion comprises periodic orbits of period 2*π*/*ω*_*j*_. We say that the system is *completely integrable*.

Would not it be nice, at least in a sufficiently small neighbourhood of the zero equilibrium, to find coordinate transformations to successively remove all the nonlinear terms from the equation (cubic and higher terms in *H*) so that the equation is once again trivially integrable up to any given power? Formally, such reductions can be performed using *canonical* (i.e. Hamiltonian-structure-preserving) *near-identity* (i.e. differing only in nonlinear terms) transformations; see, for example, Meyer *et al.* [17] for details.

But a naive approach can go wrong. Suppose, for example, *H*_1_ contained a term 
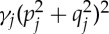
 for some *j* and some given non-zero coefficient *γ*_*i*_, leading to terms 4*γ*_*j*_*p*_*j*_(*p*_*j*_+*q*_*j*_)^2^ in the 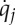 equation and 4*γ*_*j*_*q*_*j*_(*p*_*j*_+*q*_*j*_)^2^ in the 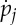 equation. Such a term is called *resonant* because, no matter what near-identity transformation is chosen, a nonlinear component of this term remains. The resonance occurs in terms that contain powers 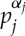 and 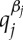, where certain integer relationships existing between the corresponding eigenvalues ±i*ω*_*j*_, *j*=1,…,*n*. The specific fourth-order expression 
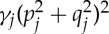
 contains resonant terms where *α*_*j*_(i*ω*_*j*_)^2^+*β*_*j*_(−i*ω*_*j*_)^2^=0 with *α*_*j*_=*β*_*j*_=2. Other resonances can arise due to internal resonances where two or more of the frequencies *ω*_*j*_ are rationally related (which leads to the so-called *small divisors* problem in Hamiltonian systems that had greatly exercised Poincaré and his contemporaries). A good choice of transformed variables are the so-called action-angle coordinates, and the resulting system is often called the *Birkhoff normal form* [18].

### (b) General first-order normal forms

The problem is that many vibration problems (1.1) cannot be written in Hamiltonian form owing to the presence of non-conservative forces due to excitation, damping, friction or gyroscopic effects. Even if a Hamiltonian formation does exist, the mechanical interpretation in terms of velocities and displacements is likely to be lost. Instead, there is a more general theory of normal forms that can be applied to arbitrary-dimensional dynamical systems of the form
2.2
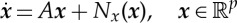
in the neighbourhood of an equilibrium point, where *N*_*x*_ represents nonlinear terms (with the subscript reminding us that we are in the original unscaled coordinates ***x***). Here, we shall be interested in the case that the Jacobian is non-hyperbolic (that is, there are eigenvalues on the imaginary axis). Often this theory is implemented in conjunction with centre manifold theory so that only the nonlinear terms associated with the non-hyperbolic degrees of freedom are retained [4,19].

Note that the unforced version of (1.1) can be written in this form by setting the number of states *p*=2*n* and
2.3
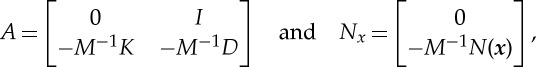
in which 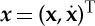, as pointed out by Jezequel & Lamarque [7].

Now, let us suppose that a linear transformation ***x***=*Φ***q** is applied to (2.2) so that it is written in the form
2.4
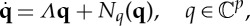
where *N*_*q*_(**q**)=*Φ*^−1^*N*_*x*_(*Φ***q**) and *Λ* contains the linearization in the simplest (Jordan canonical) form.

We seek to systematically change variables by a sequence of near-identity transformations to remove non-resonant terms of successively higher powers in the Taylor series expansion of the nonlinear term *N*_*q*_. That is, we seek a new variable 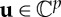 with
2.5
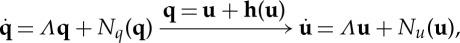
where **h**=*o*(**u**), and *N*_*u*_ contains only resonant terms (in a sense to be made precise shortly). To find the unknown function **h**(**u**), we differentiate the transform, 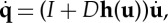 and substitute for 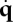 and 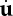 to give
2.6
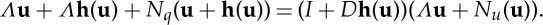
On its own, (2.6) is a complicated, nonlinear functional equation for **h**(**u**). But we simplify it by expanding **h**(**u**) as a Taylor series and solve, where possible, for the unknown coefficients of **h**, term by term. Thus, let **h**(**u**)=**h**^(2)^(**u**)+**h**^(3)^(**u**)+**h**^(4)^(**u**)+⋯ , where each **h**^(*k*)^ is a sum of homogeneous monomial terms of degree *k*. That is, the *i*th component of **h**^(*k*)^ takes the form
2.7
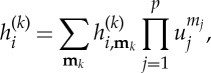
where we use a vector notation for multi-indices such that **m**_*k*_=(*m*_1_,*m*_2_,*m*_3_,…*m*_*p*_), where *m*_*j*_ is a whole number in the range 0≤*m*_*j*_≤*k* with the additional condition that 
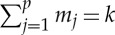
 and the sum in (2.7) is over the set of all such indices. So, for example, for *p*=2 and *k*=2, we have **m**_*k*_=(2,0), (1,1) or (0,2) such that

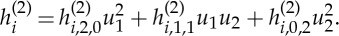


Now we are in a position to simplify (2.6). Suppose that coordinate transformations have taken place to remove all non-resonant terms up to *k*−1. Then, at 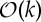, (2.6) reads

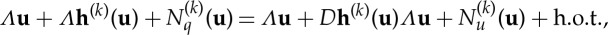
where again a superscript (*k*) on *N*_*q*_ and *N*_*u*_ indicates terms that are homogeneous polynomials of degree *k* and the terms involve (*k*+1)-powers and higher. This expression can be rearranged to read
2.8
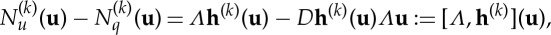
which is known as the *homological equation* associated with the linear operator *Λ*. The operator [⋅,⋅] on the right-hand side is known as the *Lie bracket* between *Λ* and **h** (which is closely related to the so-called *Poisson bracket* in the case that the system is Hamiltonian).

It might be worthwhile to remind ourselves what we are trying to do. We want to choose the coefficients of all the terms in **h**^(*k*)^ to make 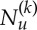 as simple as possible. Note that the Lie bracket can be thought of as a linear operator acting on the set of homogeneous polynomials of degree *k*. If this linear operator is invertible, then the homological equation will have a unique solution for any choice of 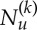. In particular, we are free to choose 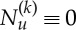, and then we will have found a unique solution for the coefficients of **h**^(*k*)^ to make all the *k*th power terms disappear in the simplified system. Terms that cannot be removed in 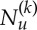, the resonant terms, arise precisely when the Lie bracket is non-invertible.

Note that the Lie bracket as an operator depends on the matrix *Λ*, hence this matrix must contain all information necessary to define which terms, in the space of homogeneous polynomials of degree *k*, are resonant. To spell out which these terms are, it is most convenient to look at the homological equation (2.8) term by term to end up with an *indicial* form of the equation in terms of the indices *m*_*j*_.

The indicial equation can be derived in complete generality for any Jordan canonical form of the matrix *Λ* [6]. But for ease of explanation, we shall treat the simplified case where the eigenvalues of *A* are *semi-simple*. That is, the geometric multiplicity of each eigenvalue is equal to its algebraic multiplicity, and hence *Λ* is *diagonalizable* so that
2.9
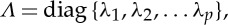
where 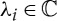 are the eigenvalues of *Λ* allowing for multiplicity. Note how the unforced general vibration system (2.3) can be transformed using eigenvectors into (2.4) with *Λ* of the form (2.9) with (λ_2*i*−1_,λ_2*i*_)=(+*ω*_*i*_,−*ω*_*i*_), for *i*=1,…*n*.

Under the assumption (2.9), the indicial form of the homological equation reads
2.10
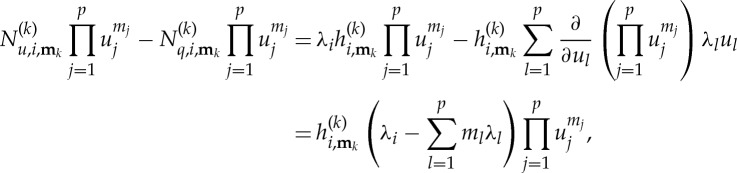
where a subscript (*i*,**m**_*k*_) on *N*_*q*_ and *N*_*u*_ represents the corresponding coefficient of the *k*th power term with vector index **m**_*k*_.

Looking at the form of equation (2.10), we find that we are free to choose 
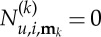
 and
2.11
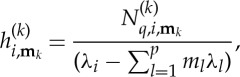
thus removing the 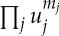 term from the simplified equation (the normal form), unless
2.12
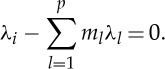
The condition (2.12) is thus precisely the condition for 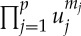 to be a resonant term of the *i*th equation. For resonant terms, we instead choose 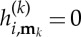 and then 
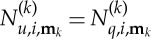
 and this term remains in the simplified normal form.

The normal form method then carries out the above procedure successively for each value of *k*, starting with *k*=2. For each *k*-value, we step over all possible indices {*i*,**m**_*k*_} and choose the coefficient 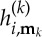 to satisfy (2.11), unless {*i*,**m**_*k*_} satisfies the resonance condition (2.12) in which case we choose 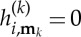. Note that at each level *k*, the near-identity transformations used to remove the non-resonant terms introduces new terms with powers greater than *k*, so before proceeding to level *k*+1, the new system must be fully calculated. The value of *k*>2 at which we stop this process is called the *degree* of the normal form. Such normal forms are also sometimes called *truncated normal forms*.

### (c) Unfolding, truncation and dynamics of the normal forms

The notion of a normal form is often applied to situations where there is a local bifurcation of the equilibrium point **q**=**0** (e.g. [19] or [4]). In this case, we think of the system (2.4) as depending on parameters 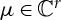 (typically the parameters are real, but they need not be) and *r* corresponds to the codimension of the bifurcation which occurs at *μ*=0. We then define an extended set of unknowns 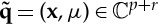 with the additional *r* trivial equations 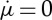. Thus, we get
2.13
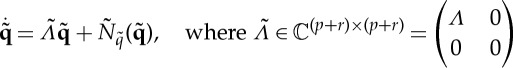
and all *μ*-dependence appears inside the first *p* components of the new nonlinear term 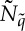. The final *r* components of 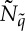 are precisely zero. The normal form method now proceeds exactly as defined above with *p* everywhere replaced by *p*+*r*.

We can then apply the normal form method as outlined above to the system (2.13). The resulting normal form is called an *unfolded normal form*. Sometimes judicious choices can be made for certain coefficients of a normal form, or by scaling arguments certain coefficients can be set to unity or zero. Such systems are sometimes called *hyper-normal forms* [6].

The normal form provides a way of simplifying the dynamics of a system near a non-hyperbolic equilibrium, and to classify the dynamics as belonging to one of a few pre-analysed cases according to the sign of certain critical coefficients in the normal form. One of the problems with using normal forms to provide more precise details is that, in general, they represent an asymptotic expansion only. Hence, regardless to which degree *k* they are truncated, they ignore the *beyond-all-orders* terms which can lead, for example, to transverse homoclinic and heteroclinic tangles. These will never be captured by the normal form, see, for example, Champneys & Kirk [20] and references therein for the case of the normal form of the codimension-two saddle-node/Hopf bifurcation (also known as the Gavrilov–Guckenheimer bifurcation). Occasionally (roughly speaking, when the dynamics of the normal form does not include any structurally unstable homo-/heteroclinic connections), an unfolded, truncated (hyper)-normal form can be shown to contain dynamics that are topologically equivalent to all possible structurally stable dynamics that can occur in a neighbourhood of the bifurcation point. Such a normal form is called a *versal unfolding* [21] or a *topological normal form* [19], an example of which is the normal form of a Hopf bifurcation, truncated after third-degree terms.

Consider the damped Duffing equation
2.14
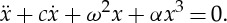
We can include this in the normal form via an additional equation 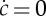. Carrying out the above steps, we first diagonalize the linear part by writing

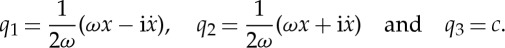
Then, we end up with a system of the form (2.13) for **q**=[*q*_1_,*q*_2_,*q*_3_]^T^ with

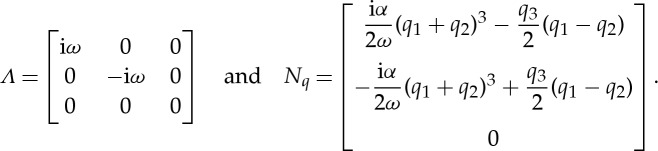
Now, from the form of the linearization of the extended system up to 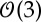, we see from (2.12) that quadratic terms of the form *q*_3_*q*_*i*_ are resonant, as are cubic terms of the form 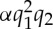 in the 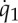 equation or of the form 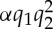 in the 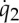 one. We can remove all non-resonant cubic terms with a transformation of the form

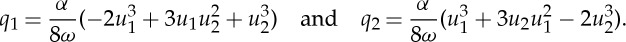
Hence, the third-degree normal form of the damped, unforced Duffing equation can be written as
2.15
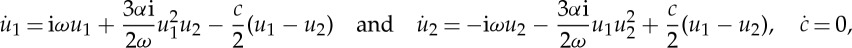
cf. Jezequel & Lamarque [7].

Jezequel & Lamarque [7] extended this normal form method in combination with the harmonic balance method to consider forced vibration problems of the form (1.1). This enabled them to predict resonant responses in the case that the forcing frequency *Ω* is close to one of the natural frequencies of the system. There remains a philosophical weakness that their method does not produce an approximation to the system itself, as Poincaré’s envisaged, it simply uses the normal form method to predict resonant (periodic) responses within a harmonic balance framework. This does not allow the complete dynamics close to certain multiple resonances to be explored, which in general will include quasi-periodic and possibly chaotic motion.

## 3. Normal forms for second-order mechanical systems

One of the weaknesses of using the general normal form method for vibration problems, as in Jezequel & Lamarque [7], is that this does not use the specific structure of the matrix *A* in (2.3). In view of this, Neild & Wagg [8] produced an extension to the method that can be applied directly to second-order differential equations of the form (1.1), preserving the decomposition into velocity and position variables. We term this the *second-order normal forms*, referring to the order of the differential equations rather than the level of accuracy achieved. As shown in §3c, this can lead to more accurate predictions of resonant amplitudes. However, their method still relies on the harmonic balance method and so one cannot appeal directly to the mathematical theory of normal forms. The key contribution of this paper then is to show this second-order normal form method can be re-derived in a similar spirit to the analysis of the previous section.

### (a) Theoretical development

Consider a system of the form (1.1). We shall specifically treat the case where *r*(*t*)=e^i*Ωt*^ for some fixed forcing frequency *Ω*, although the method is easily generalizable to quasi-periodic forcing. The first step is to diagonalize the system as much as possible. To do this, we set **x**=*Φ***q**, where *Φ* is a matrix of eigenvectors of *M*^−1^*K*, giving

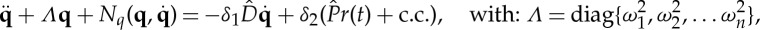
and where 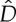, 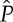 and *N*_*q*_ are the transformed damping matrix, forcing vector and nonlinearity, respectively, and *ω*_*i*_ are the linear frequencies of the system. Here, we have introduced two small parameters *δ*_1_ and *δ*_2_ which will play the role of unfolding parameters. Furthermore, we shall be interested in the critical situation where there is a resonance between the forcing frequency and one of the natural, undamped frequencies of the system, without loss of generality, *ω*_1_. That is, we assume that *Ω*=*ω*_1_+*δ*_3_, where *δ*_3_ is a third unfolding parameter. Finally, we suppose that there are no other linear resonances when ***δ***=(*δ*_1_,*δ*_2_,*δ*_3_)=**0**; that is *Ω*≠*ω*_*i*_ for *i*=2,…*n*. Then, we can perform a further linear change of variables to remove the non-resonant forcing terms by writing
3.1
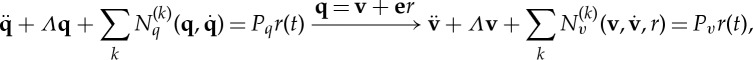
where *e*_1_=0 and 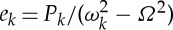 so that the forcing term is in the first, resonant equation only. Here, nonlinear terms have been expressed in monomial form.

We shall now use a tilde to represent an extended variable, so that 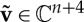 is the vector (**v**,*r*,***δ***) and write the complete system in the form
3.2
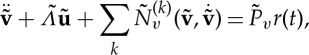
where the first *n* components of 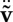 are given by the transformed equation in (3.1) and the last four components by 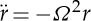 and 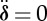. Here, the first *n* components of 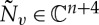 are equal to the nonlinearity *N*_*v*_ and the last four components are zero. Also,
3.3
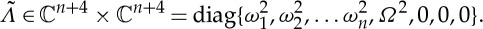
Note that forcing, damping and frequency mismatch are all now thought of as nonlinear terms, because they contain terms that involve an unfolding parameter *δ*_*i*_ times a state variable *u* or *r* or their derivatives, and we think of the *δ*s as state variables too. Observe how the system (3.2) looks remarkably similar to the unfolded first-order normal form (2.13) apart from the second derivatives on the left-hand side. This difference will be important when we try to compute the normal form for such a system.

Now we focus on transforming the equations of motion to the form
3.4
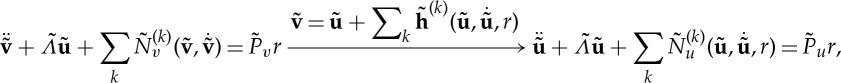
where, as with 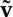, 
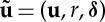
. As before, we suppose that all non-resonant nonlinear terms have been removed up to degree *k*−1 using the transformation to remove terms at 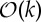. Eliminating 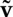 leads to the 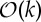 equation
3.5
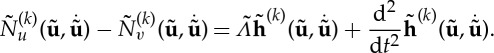


To proceed, we next complexify the system by writing
3.6
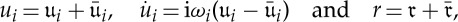
where *u*_*i*_ and 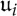 are the *i*th elements in **u** and 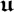, respectively, and the bar indicates the complex conjugate. This results in 2*n*+5 arguments for 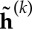, 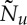 and 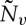 which we temporarily call **z** such that 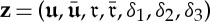. As with the first-order case, we wish to write (3.5) in indicial form and solve the corresponding homological equation. To do this, we again assume a particular component for the pre- and post-transformed nonlinear terms and the transform term of
3.7
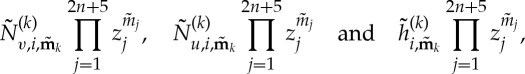
respectively. Here, we define the augmented vector 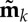 of length 2*n*+5, corresponding to the 2*n*+5 arguments, **z**, and may be written using the notation 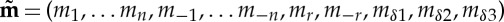, with the *j*th element referred to as 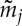. Using this indicial representation of the *n*+4 element vectors 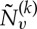, 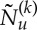 and 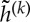, (3.5) may be written in homological form as
3.8
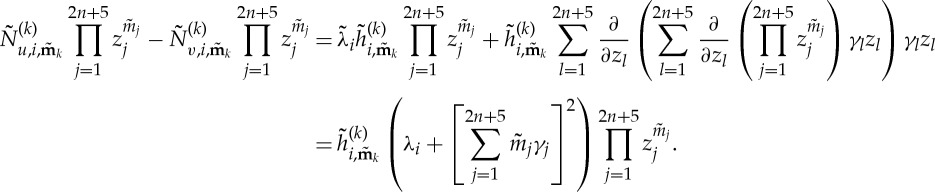
Here, 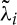 is the *i*th diagonal element in 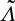, see (3.3), and 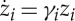 such that *γ*_*i*_ is given by
3.9
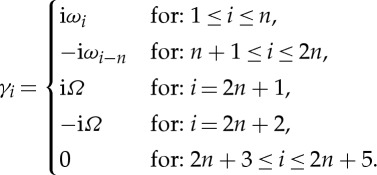
By considering the form of 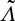 and *γ*_*i*_, equation (3.8) can be simplified to give for 1≤*i*≤*n*
3.10
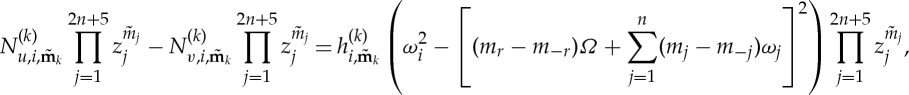
where, for these values of *i*, 
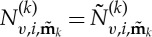
 and we define 
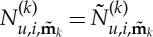
 and 
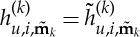
. For *n*+1≤*i*≤*n*+4, 
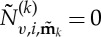
, so we set 
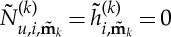
.

The homological equation can be inverted and solved for 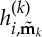 provided we avoid the resonant terms for which
3.11
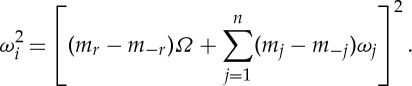
Note that *m*_*r*_ or *m*_−*r*_=1 with *m*_*δ*,1_=1 and *i*=1 automatically leads to resonance because of the fundamental resonance we have assumed between the forcing and natural frequency *ω*_1_; that is, *ω*_1_=*Ω*. But there will in general be other resonances; for example, whenever *m*_*i*_=*m*_−*i*_+1 as with the normal form of the Duffing equation. As we shall see in later examples, there can be further *internal resonances* if *kω*_*i*_=*lω*_*j*_ for some integers *i*, *j*, *k* and *l*.

### (b) Practical implementation

Having shown in principle that a second-order version of the normal form can be derived for structural vibration systems, we now turn to a practical implementation of it, as discussed in detail in Neild & Wagg [8] and Neild [22]. This implementation avoids the need to explicitly form the extended 2*n*+5 dimensional state vectors. The key difference in this implementation is that, rather than seeking a specific normal form, we seek to project the system onto a nonlinear equivalent of normal modes, in a harmonic-balance-like approach. As we shall see this approach relies on computation of exactly the same normal-form coefficients as in the previous subsection. So, we specifically suppose that in the complexification step (3.6), we seek a solution of the form

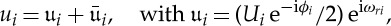
where *ω*_*ri*_ is the *response frequency* in the *i*th modal coordinate direction. Here, *ω*_*ri*_ is, in general, a nonlinear function of the variables *u* that must be determined as part of the calculation. It corresponds, for an unforced system to the *nonlinear natural frequency* for moderate amplitude periodic orbits. This concept can be made precise using the Liapunov centre theorem *ω*_*ri*_=2*π*/*T*, where *T* is the period for a given amplitude periodic orbit in the NNM invariant manifold (in the sense of Shaw & Pierre [9]) tangent to the *ω*_*i*_ eigenspaces. In the presence of forcing, the response frequency of a resonant mode is taken to be *ω*_*ri*_=*Ω*.

Now the analysis is the same as before using (3.2) and then the equivalent to (3.1), but without using the extended states. The equivalent of the expression (3.5) for the *k*=1 case reads
3.12
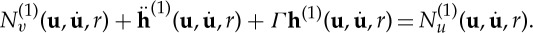
Here, we have introduced diagonal matrix *Γ* with the *i*th diagonal element being 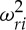. To first approximation, *k*=1, this equals *Λ* as 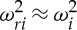. Making this substitution has no algebraic effect on the lowest degree approximation to the normal form, but results in more accurate prediction of the harmonics of the responses [23]. As a consequence, the *k*>1 equations contain *Γ*−*Λ* correction terms (although an acceptable approximation is normally achieved without evaluating these equations).

As before, after complexification of the equations, we now seek to solve (3.12). This can be for each of the terms in the 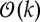 nonlinearity 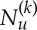. Now, instead of introducing multi-indices {*i*,**m**_*k*_}, we simply place all the combinations of states that exist in *N*^(*k*)^ into vector u* (of length ℓ) and introduce coefficient matrices h*, n* and 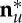 in 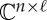, so that
3.13
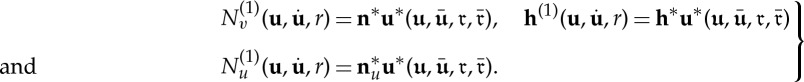
This is an alternative to the arbitrary product notation used earlier, see (3.7) for the indicial form of this. Using the new representation, (3.12) may be rewritten as
3.14
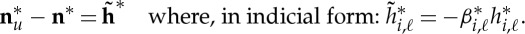
Considering the indicial form, it can be shown that
3.15
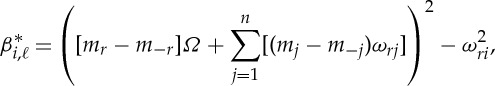
where the form of the ℓth term in u* is given by
3.16
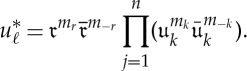
Noting the minus sign in (3.14), introduced to maintain consistency of the definition of β* with previous publications, (3.15) is exactly equivalent to (3.10). The appearance of *ω*_*ri*_ rather than *ω*_*i*_ is due to the introduction of *Γ*.

Considering the indicial version of (3.14), 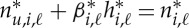, if 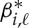 is zero, it is *resonant* and must be retained in the post-transformed nonlinear vector, otherwise the nonlinear term can be removed using the transform such that
3.17
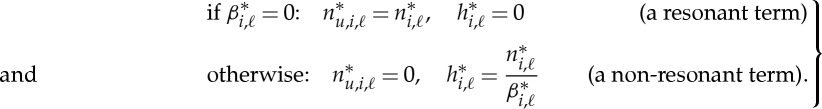


Having identified h* and 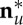, and hence **h**^(1)^ and 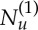 using (3.13), we can approximate the transform and transformed dynamic equations to
3.18
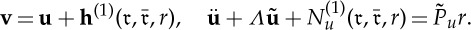
The approximation here is that we have used only the *k*=1 terms, **h**^(1)^ and 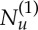 rather than a summation over all *k* as was used in (3.4). To refine these expressions, the higher *k* terms can be included using a similar approach (e.g. [23]), however this is not normally necessary.

In the case of forced systems, when the forcing is close to resonance for all the modes, the forcing transform (3.1) reduces to **q**=**v** and the nonlinear transform 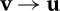 remains the same as the unforced case, this is discussed further in the example that follows. When non-resonant forcing occurs in any mode then additional terms appear in u* due to the forcing transform—for examples which include non-resonant forcing, see Wagg & Neild [24] and Neild & Wagg [8], in which a two-mode forced system is assessed. Wagg & Neild [24] also contains a discussion and comparison of the second-order normal forms alongside the harmonic balance, averaging and multiple scales techniques for the vibration of a forced single mode system. The relationship between the forced response and the NNMs of the unforced system is discussed in Hill *et al.* [25].

Two examples are now considered, the first is the Duffing oscillator which will be used to compare results from the first-order, state-space, variant of the normal forms with the second-order, oscillator equation, variant. Both responses will be considered using just the *k*=1 terms. This example will also be used to demonstrate the forced and damped case. The second example considers the NNMs for a two-mode model of a cable under free vibration. Both the resonant and the harmonic responses are calculated to yield the response frequencies and NNM mode-shapes.

### (c) Example: the Duffing equation

Let us consider the unforced, undamped Duffing equation
3.19
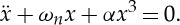
As this system has one degree-of-freedom *x*=**x**=**q**=**v**. Using the matrix formulation, we keep the cubic term *αx*^3^, so that 
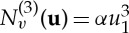
. Using (3.13), n* and u* may be defined, then β* can be calculated using (3.15) and from this the transformed nonlinear terms and the transform can be found using (3.17). This gives

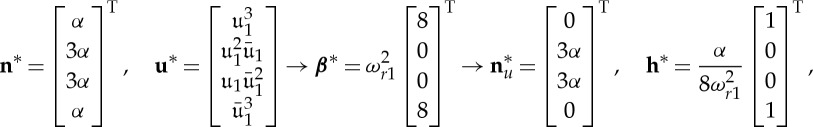
Using (3.1), (3.4) and (3.13) allows the transformed equation of motion along with the transform to be written as

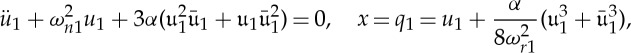
respectively. As the response for the *i*th mode will just be at the resonant frequency *ω*_*ri*_, we can write the steady-state solution
3.20
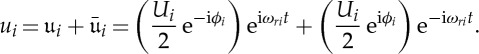
Hence,
3.21
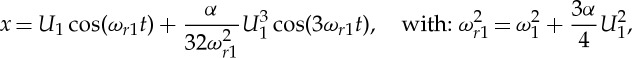
where we have defined *t* such that *ϕ*_1_=0. Note that as the harmonic terms were removed using the near-identify transform, *U*_1_ conveniently represents the amplitude of the resonant response.

Considering the case where the system is damped and forced at a frequency close to resonance, such that 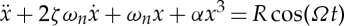, the forcing transform is **q**=**u**. As the forcing is near-resonant, the response frequency is set as *ω*_*r*1_=*Ω* and the near-identity transform is the same as that derived above, it is unaffected by the introduction of the forcing or the damping (which can be thought of as a resonant term). The resulting resonant dynamics and transform are

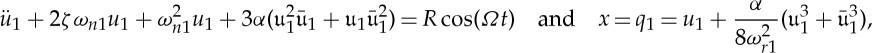
respectively. Using the steady-state solution (3.20) and balancing the complex exponential terms allows the amplitude relationship 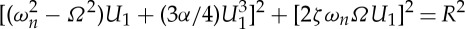 and phase relationship 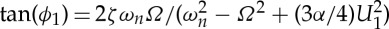 to be found. Once *U*_1_ and *ϕ*_1_ have been found for a given forcing, then the response, including harmonics, can be calculated using the transform equation and (3.20).

The unforced solution can be compared with the solution derived using the first-order version of the normal form technique in which the state-space, rather than oscillator, form of the equations are used. Here, the first step is to rewrite the equation of motion in state-space form using 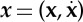 and then to apply a linear transform giving

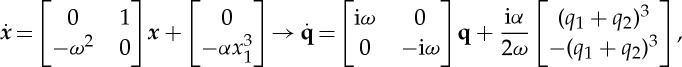
where, as the system is written in first-order form, we have

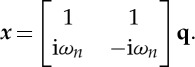


Applying the normal form transformation in a similar way to the second-order matrix formulation, now using 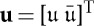 gives
3.22
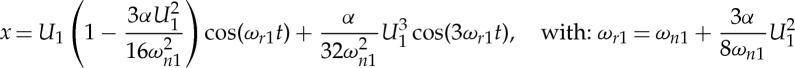
[7,12]. Note that here *U*_1_ does not fully represent the amplitude of the resonant response. This is because, when identifying the transform, only the e^i*ω*_*r*1_*t*^ terms are kept in the first equation of motion in **U** with e^−i*ω*_*r*1_*t*^ terms being represented in the transform and vice versa for the second equation of motion [8].

**Figure 2.**
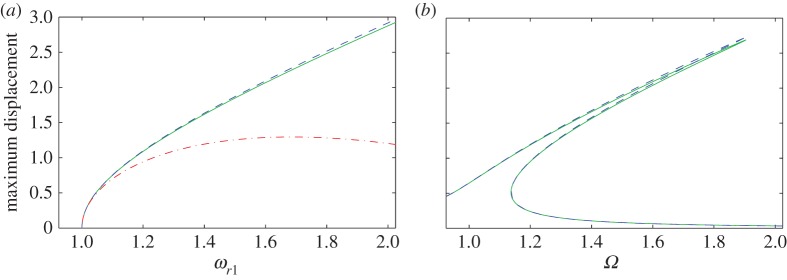
Duffing oscillator example with *ω*=1 and *α*=0.5: (*a*) backbone curves and (*b*) forced response curves when *ζ*=0.01 and *R*=0.1. The solid line shows the exact computation using AUTO; the dashed line shows the result of the second-order normal form calculation; and the dashed-dotted line the result of the first-order normal form.

The response (or backbone) curves for both the second- and the first-order variants of the normal forms, (3.21) and (3.22), respectively, are shown in figure 2*a*. For comparison, the solution derived using numerical continuation, using AUTO [26], in which no assumptions are made regarding the smallness of terms, is also shown. It can be seen that at low amplitudes, corresponding to weaker nonlinear terms, there is good agreement in the prediction of the response frequency, however as the amplitude increases the higher power terms become significant, firstly for the first-order normal form approximation at about 0.6 and then also for the second-order version at about 1.8. Figure 2*b* compares the second-order normal form and the AUTO predictions of a forced response, again it can be seen that good agreement is achieved.

## 4. Application to nonlinear normal modes

We now consider how the application of normal forms to unforced, undamped second-order differential equations can be used to calculate the NNMs of a system. Second-order normal forms can be used to derive the backbone curves of a system via the resonant responses (e.g. Wagg & Neild [24] or Hill *et al.* [27]). Here, we extend this to finding the NNM mode shapes, which requires both the resonant response and also the harmonics captured in **h**. While the technique is general, to facilitate the discussion we apply it to a well-known nonlinear system—the dynamics of a taut cable. Specifically, we will consider the interactions between the first out-of- and in-plane modes (we use mode to indicate the modes of the linearized system). In doing this, to calculate the associated NNMs, we will derive the harmonics excited in *all* modes due to resonant responses in the first out-of- and in-plane modes.

To examine the dynamics of a taut cable, we use the modal equations of motion derived by Warnitchai *et al.* [28] and discussed in detail in Wagg & Neild [24], which accounts for gravitational sag and tension effects. The motion of the cable out-of- and in-plane (where in-plane relates to the plane in which gravitational sag occurs) are represented as
4.1
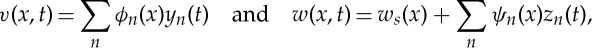
where *x* is the distance from one of the supports and the other support is positioned at *x*=ℓ. Here, *ϕ*_*n*_(*x*) and *ψ*_*n*_(*x*) are the *n*th out-of- and in-plane linear mode shapes, respectively, *y*_*n*_(*t*) and *z*_*n*_(*t*) represent the modal contributions for the *n*th modes and *w*_*s*_(*x*) captures the static sag in the cable (noting that *v* and *w* are defined as zero on the chord line between the supports). The dynamics for the *n*th out-of- and in-plane modes may be expressed as
4.2
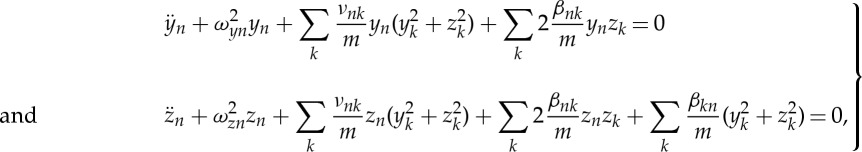
respectively. Here, modal damping and external forcing terms have been removed from the original derivation in line with investigating the backbone curves of the system. Parameters *m*, *β*_*ij*_ and *ν*_*ij*_ are given in Warnitchai *et al.* [28] and Gonzalez-Buelga *et al.* [29], but importantly *β*_*ij*_ is zero for even *j*. The natural frequencies of the out-of-plane modes are proportional to the mode number *n*, and for even *n*, natural frequencies for the in-plane modes match those of the out-of-plane ones. For odd *n*, the in-plane natural frequencies are slightly higher than the out-of-plane ones, again see [28,29]. The in-plane axis is defined as positive down, and so gravitational sag is positive.

Gonzalez-Buelga *et al.* [29] considered these equations in terms of internal resonance showing that 1 : 1 resonance occurs between the second out-of- and in-plane modes and Macdonald *et al.* [30] generalized this for the *n*th out-of- and in-plane modes. In addition, they both show that 2 : 1 resonance can occur, but only for the case where the cable is inclined. Hill *et al.* [27] identified the backbone curves when the system is reduced to the first out-of- and in-plane modes. Here, we build on this work by deriving algebraic expressions for the NNMs associated with these backbone curves, which requires not only the resonant responses of the two modes but also the harmonic response in all modes. To do this, we first calculate the backbone curves along with expressions that capture the harmonics contained in the response. These are then used to find the NNMs in terms of just the two first modes and finally the additional modal contribution due to harmonics in other modes is added to give the full NNM expressions.

### (a) Resonant equation of motion and harmonic response

Letting **q**=(*y*_1_ *z*_1_)^T^ the modal equation of motion for the reduced two-mode model may be written in the form 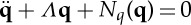, an unforced version of the left-hand expression in (3.1), where

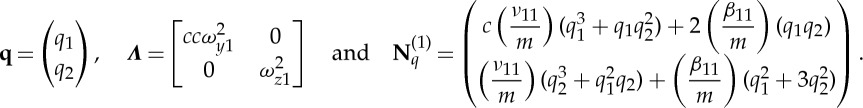
Here, taking 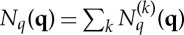, all the nonlinear terms have been placed in 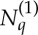. As there is no forcing, **v**=**q** and so 
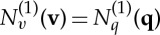
. To calculate the nonlinear transform, see (3.4), 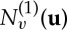 is considered. Rewriting this nonlinear vector in terms of 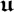, where, for the *k*th coordinate 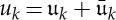, gives

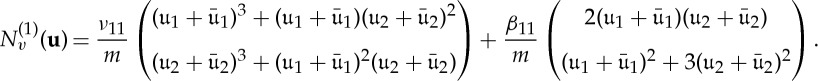
This can now be expressed in matrix form 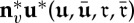, see (3.13) along with (3.6), and the matrix β* can be derived using (3.15), where it can be assumed that, due to the closeness of the natural frequencies, we can write the response frequencies as *ω*_*r*1_=*ω*_*r*2_. Using (3.17), the nonlinear vector in the transformed equation of motion and the transformation vector can be written as
4.3
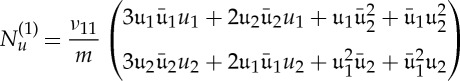
and
4.4
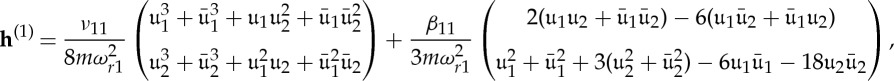
respectively. The modal response of the system can now be written as **q**=**v**=**u**+**h**^(1)^, where **h**^(1)^ captures the harmonic content of the response.

Using the steady-state solution (3.20), the transformed equations of motion, 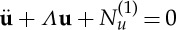, can be written as
4.5
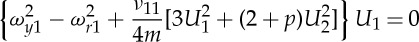
and
4.6
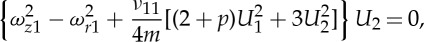
where 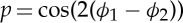 with the condition 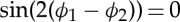 to ensure (4.3) is real. This allows possible solutions *p*=±1. It can be shown that only *p*=−1 gives physically meaningful solutions (as *ω*_*z*1_≠*ω*_*y*1_) which means that when both linear modes are present, they are ±90° out-of-phase, see Hill *et al.* [27] for a more detailed discussion of this.

### (b) Backbone curves and modal response

**Figure 3.**
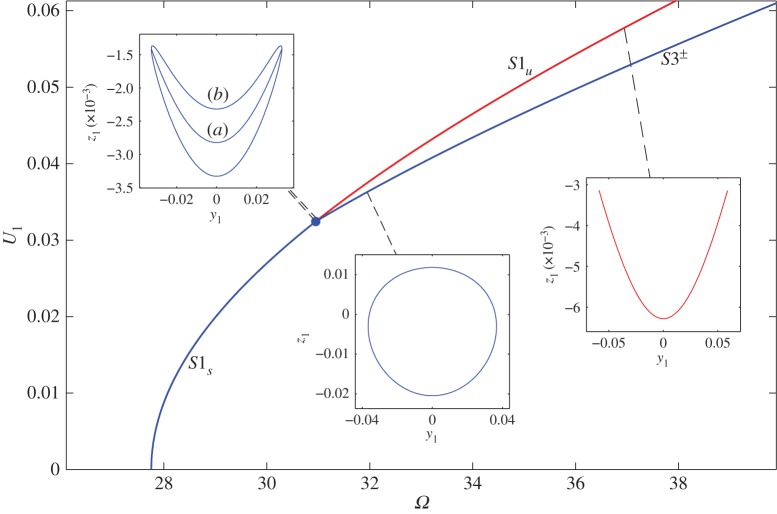
Backbone curves *S*1 and *S*3^±^ for a cable with inset panels showing the motion in projection *q*_1_=*y*_1_ versus *q*_2_=*z*_1_. The inset panel for the response near the bifurcation contains two responses; the response line (*a*) corresponding to a point on *S*1_*s*_ just below the bifurcation and the response loop (*b*) corresponding to a point on *S*3^+^ just above the bifurcation. Subscripts *s* and *u* indicate that backbone curve *S*1 is stable below and unstable above the bifurcation.

Taking the physically meaningful *p*=−1 case, there are two semi-trivial solutions for (4.5) and (4.6) in which only one of the two linear modes is resonant
4.7
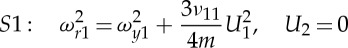
and
4.8
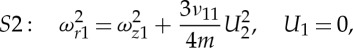
and two further solutions exist in which both linear modes are resonant
4.9
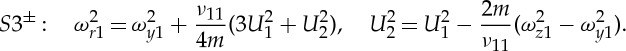
As *ω*_*z*1_>*ω*_*y*1_, it can be seen that valid solutions for *S*3 only exist if 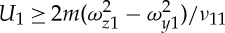. The point at which 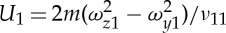 results in *U*_2_=0 and lies on backbone curve *S*1, see (4.8). Hence these *S*3 solutions (there are two solutions relating to the relative phase of the two linear modes being ±90°, solutions *S*3^±^) are branches from *S*1 following a bifurcation at *U*_2_=0. This bifurcation from the *S*1 solution is shown in figure 3 using the parameters defined in Gonzalez-Buelga *et al.* [29]. Note that the *S*3^±^ backbone curves lie on top of each other in this projection.

Using the transform equation, **q**=**v**=**u**+**h**^(1)^, and the steady-state solution (3.20) gives the response of the modal coordinates
4.10
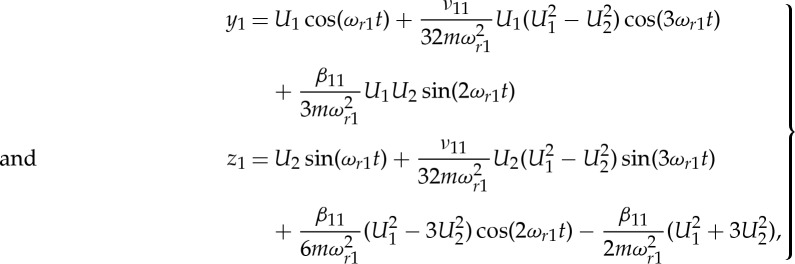
when the phase difference is *ϕ*_2_−*ϕ*_1_=90° and *ϕ*_1_ has been set to zero.

The *S*2 solution consists of purely in-plane resonant motion as *U*_1_=0. In this case, the response given by (4.10) reduces to purely in-plane motion. Harmonics exist in this motion including a static displacement which, as it is negative, reduces the apparent sag of the cable during resonance. For the *S*1 solution, which is resonant purely in the out-of-plane direction as *U*_2_=0, there are also harmonic components in the out-of-plane response. In addition, there is an amplitude-dependent in-plane non-resonant response. For the *S*1 solution example of the motion of the mid-span of the cable (where the mode shapes are unity) in the *y*_1_ versus *z*_1_ plane are shown as in figure 3 as inset plots. Note that in contrast to Hill *et al.* [27], these inset panels include the harmonic components as well as the resonant ones. Examples of the more complex motion of the *S*3 solution, in which there is 90° out-of-phase resonant motion in both planes, are also shown in figure 3. Here, it can be seen that away from the bifurcation (indicated with a dot), the cable is whirling in a near elliptical shape—it would be exactly circular if *ω*_*y*1_=*ω*_*z*1_, albeit offset from the origin in the vertical direction.

These inset panels show the motion at the mid-span assuming that the harmonics of the cable response are limited to the first out-of- and in-plane modes. Once additional harmonics from other modes are included in the response, the full NNM response can be derived. This is done in §4*c* for the NNMs that contain resonant responses solely in either or in both the first out-of- or in-plane modes.

### (c) Additional modal contributions

In the previous analysis, only the first out-of- and in-plane modes have been considered. It has been shown that in-plane motion is present throughout (figure 3), even for the *S*1 solution, where there is no resonant in-plane response, but there are twice- and zero-frequency components. By inspection of (4.2), it can be seen that this is due to the term 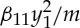 that arises from the 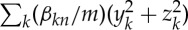 term which is due to the variation in tension in the cable during oscillations. For the *S*1 solution, where the first in-plane mode is non-resonant, this term may loosely be thought of as a non-resonant ‘forcing’ of the in-plane mode giving rise to twice- and zero-frequency response components—being non-resonant components they appear in **h**^(1)^, (4.4), as 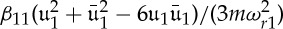.

We now assess whether non-resonant response terms can occur in higher modes of the cable due to the resonant response of the first out-of- and in-plane modes. For the *n*th out-of-plane mode, all the terms in the equation of motion (4.2) are multiples of *y*_*n*_, hence there can only be a response if the mode is resonant. For the in-plane modes, a response is possible due to the 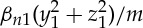 terms from the summation 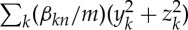. These terms can result in a non-zero **h**^(1)^, even with zero resonant response in the *n*th in-plane mode, that is captured by
4.11
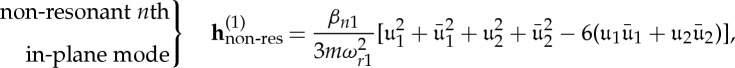
following the same approach as was used to calculate (4.4) and then setting 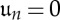. Applying the resonant response solution for the first in- and out-of-plane modes using (3.20), noting that *β*_1*n*_=*β*_11_/*n* for odd *n* and zero for even *n*, and applying the phase condition that *ϕ*_2_−*ϕ*_1_=90°, and *ϕ*_1_=0, gives the response for the *n*th in-plane mode
4.12
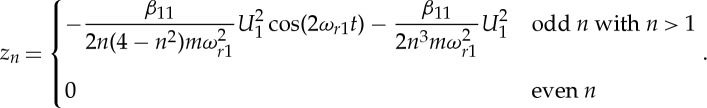
Here, we have noted that, when *n*>1, *z*_*n*_=**h**^(1)^_non-res_ if only the resonant response of the first-in- and out-of-plane modes are considered.

### (d) Nonlinear normal modes

These modal contributions can be added to those from the first-in- and out-of-plane modes, (4.10). Using (4.1), along with the mode shapes, the NNMs can be identified in terms of the lateral displacement *v*(*x*,*y*) and the vertical displacement *w*(*x*,*y*). By approximating the in-plane modes as sinusoidal, an acceptable approximation if the cable is taut [24], the response may be written in terms of amplitude of the resonant responses *U*_1_ and *U*_2_ as
4.13
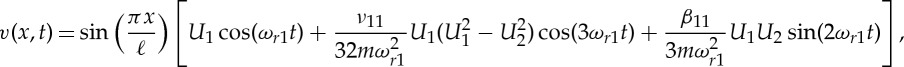
in the out-of-plane direction and
4.14
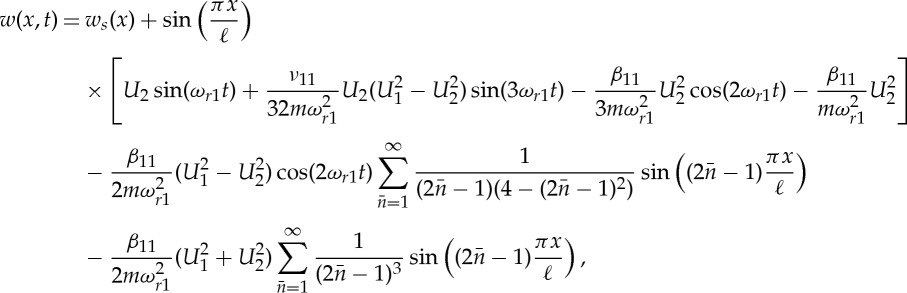
in the in-plane direction. These expressions along with the response frequency and resonant amplitude relationships, (4.7), (4.8) and (4.9), define the NNMs.

In the out-of-plane direction, the NNM mode shape is straightforward, a half sine wave matching the linear mode shape; however, the response contains not only the response frequency but also harmonics. In the in-plane direction, the NNM is more complex with modal components from all the odd in-plane modes. This expression can be significantly simplified using the Fourier series expansions

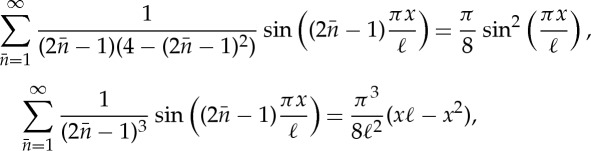
such that
4.15
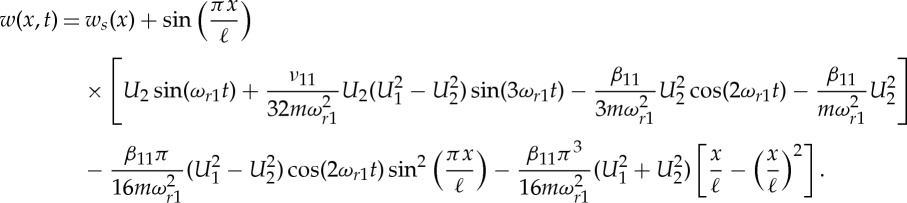
It is, perhaps, interesting to note that the last term, which is not a function of time, takes the same shape along the cable as the static sag (*w*_*s*_(*x*)∝(ℓ*x*−*x*^2^) from [24]). It can therefore be seen as reducing the sag effect due to the increased average dynamic tension in the cable as it oscillates. The in-plane NNM response, at the level of accuracy of the normal form transformation, therefore consists of the linear mode shape oscillating at three frequencies along with a dynamically corrected static sag and a further term in the shape of the square of the linear mode shape. Due to this final term, the shape of the NNM in the in-plane direction is dependent on the amplitudes of the resonant responses in the first-in- and out-of-plane modes and varies over each oscillation.

**Figure 4.**
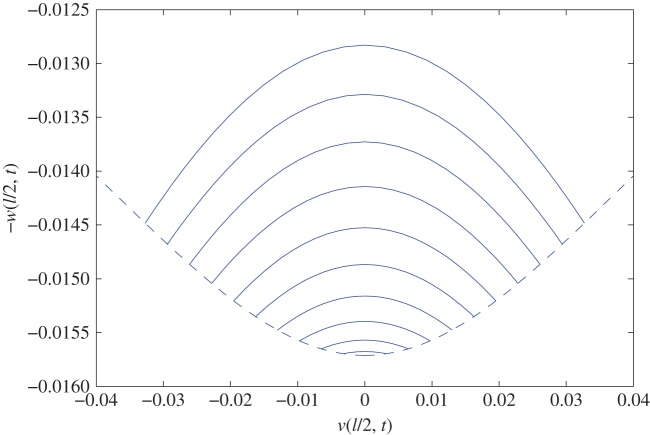
Trajectory of mid-span deflections for a range of *U*_1_ values on the *S*1 solution up to the bifurcation amplitude (solid lines) and the locus of points from which a stationary cable can be released onto a pure *S*1 solution provided the deflected shape along the length of the cable is appropriate (dashed line).

To examine these NNMs, first consider the *S*1 solution, in which *U*_2_=0. Figure 4 shows the trajectory of the mid-span deflections *v*(ℓ/2,*t*) and *w*(ℓ/2,*t*) for a range of values of *U*_1_ up to the bifurcation amplitude in the *y* versus −*z* (to account for the fact that *z* is defined positive downwards) plane using (4.13) and (4.15). For each trajectory, the resonant frequency is different, as shown in figure 3. The dashed line represents the maxima of the trajectories and corresponds to the initial deflection points from which, provided the initial shape is imposed, the system can be released into the pure *S*1 NNM. This initial shape is a half sine wave out-of-plane regardless of the amplitude of *U*_1_ but is a more complex weakly amplitude-dependent shape in-plane. Note that the in-plane deflection plotted here includes static sag—the value of which corresponds to the minimum of the dashed line where the initial out-of-plane deflection is zero. Any deflection along the NNM from this point results in the cable oscillating above the static sag position. For the *S*2 solution, as *U*_1_=0, the motion is confined to the vertical plane such that both the trajectories and the initial deflection points would lie on the vertical axis in the figure.

**Figure 5.**
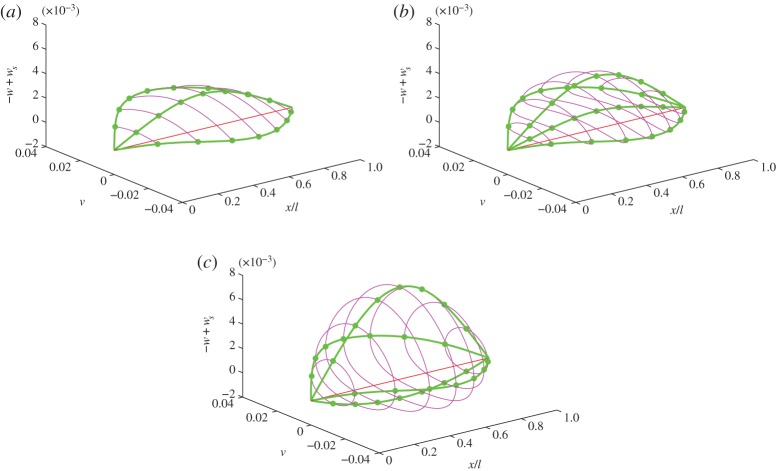
Trajectory of the cable for the *S*3 solution (*a*) at the bifurcation point and for a *U*_1_ amplitude (*b*) 0.1% and (*c*) 1% greater than that at which the bifurcation occurs. Vertical deflections are plotted relative to the static sag shape. Green lines show cable locations at quarter time period points, magenta lines shown the eighth-span traces of the cable for all time, the red line shown the sag shape and the blue line the chord between the two supports.

Considering the *S*3^±^ solution, figure 5 shows the full cable response in space when *U*_1_ is (*a*) at the bifurcation point and (*b*) at 0.1% greater than and (*c*) 1% greater than the amplitude at which the bifurcation occurs. Here, the vertical displacements are plotted relative to the static sag and hence the sag equilibrium is the straight (red) line between the supports. The cable location is shown at quarter time-period spacing as thick (green) curves and the trace of the cable over all time at eighth-span locations are shown as thin (magenta) loops. The initial transition into whirling, panel (*b*), is a solution that closely envelops the pre-whirling response, panel (*a*), as is suggested by the responses (*a*) and (*b*) in figure 3. Figure 5*a*,*b* also shows that the motion over all time occurs above the sag location. This is perhaps contrary to the expectation that whirling is initiated when the vertical momentum becomes sufficient for the cable to wrap around the zero deflection point rather like a pendulum going ‘over the top’ as it undergoes a transition from swinging to rotating motion. Instead, the initial whirling motion, panel (*b*), envelops the pre-whirling solution, and then rapidly becomes a near-elliptical orbit that encircles the sag equilibria as can be seen in panel (*c*).

## 5. Concluding remarks

The purpose of this paper has been to review the method of normal forms in the context of second-order mechanical vibration problems, to demonstrate its power, and to show how recent developments fit with the historical account of the subject. To this end, the main novel contribution is the re-derivation of the second-order normal form technique using the general theory (§3). This shows that, provided forcing and damping are considered to be weak, then forced-damped systems can be treated in a mathematically consistent way. Moreover, this treatment provides insight into the region of validity of the method. Frequency detuning, forcing amplitude and damping coefficients can be treated as *unfolding* terms of the same order of magnitude as the nonlinear terms. While this assumption of weak forcing and damping may appear restrictive, for smooth nonlinear systems close to resonance, one is usually dealing with internal forces generated from structural stiffnesses that are much larger than excitation and damping forces.

Other novelties of this work include a re-derivation of the homological equation (3.11) without reference to harmonic balance or an artificial book-keeping parameter *ε* as was used in Neild & Wagg [22]. Also, we have applied the method to the computation of the NNMs of a multiple-degree-of-freedom model of a cable close to the resonance between in-phase and out-of-phase fundamental modes. This has enabled a general expression for the responses, (4.13) and (4.15), including contributions from arbitrarily many higher order modes to be derived. These fully parametrized expressions have enabled us to draw novel insight into the mechanism of transition from planar to whirling solutions in the problem.

As discussed elsewhere, the second-order normal form method keeps the transform physically relevant. It could be argued that the concept of the normal form is the natural generalization of linear modal analysis for finite degree of freedom systems. Linear modal analysis essentially identifies eigenvectors and their associated natural frequencies and damping constants. In addition, the normal form identifies the key nonlinear terms that must be included for a complete description of the dynamics. Normal form analysis can be compared to other methods for providing approximate solutions to weakly nonlinear vibration problems. There are a wide class of perturbation methods; averaging, harmonic balance, multiple scales, etc. (e.g. [31] or [32]). These have been applied successfully by many authors to produce approximate solutions close to many different types of resonance. While the methods often give the same algebraic solutions, for example for the prediction of backbone curves in the absence of forcing and damping, it is often hard to predict the region of validity of the analysis, and they tend not to explain the full dynamics including harmonics. The normal form is philosophically different; the method works not by looking for specific kinds of solution, but by providing sequences of approximations to the equations of motion themselves. Thus, one still has access to the complete dynamics. Moreover, the method is algorithmic and can readily be carried out computationally.

It is also useful to compare normal form analysis with direct calculation of NNMs. Our application to cable dynamics in §4 has shown that the normal form provides a natural way to compute local (i.e. weak amplitude) approximations to NNMs in undamped systems. However, as argued in the caption to figure 1, NNMs are not uniquely defined in the presence of damping, which provides both conceptual and computational problems. For example, in a recent paper by Renson *et al.* [33], a finite-element method was proposed for computing NNMs directly. In example 5.2 of that paper, a large-amplitude approximation to a NNM is computed under two different assumptions on the magnitude of damping for a two-degree-of-freedom nonlinear oscillator. Not only is it found that the NNM shape depends strongly on the damping coefficient, but the NNM is only computed for the most strongly damped mode; it is not uniquely defined for the other mode. Unfortunately, in reality, the least damped mode is the one that is most likely to be excited in practice.

By contrast, the normal form method is not limited by restriction to the most heavily damped mode. Indeed, normal form methods are routinely used in conjunction with Melnikov’s method where the forcing and damping terms play the role of unfolding parameters. This enables the complete dynamics (not just that restricted to putative NNMs) to be studied in the neighbourhood of a resonance (e.g. [34]). Furthermore, in this paper, it has been shown that damping and forcing can naturally be included as unfolding parameters in the second-order normal form. Examples of the second-order normal form method applied to forced and damped vibration problems were presented in Wagg & Neild [24].

Finally, we note that the second-order normal form method is naturally generalizable to higher degrees of freedom, and to cases of multi-frequency excitation, parametric resonance and multiple or internal resonances. These will simply result in different detuning parameters *δ* in the notation of §3a. An exploration of such cases is left for future work, as is application of the method to systems of higher degrees of freedom using computational methods.
